# A facile synthesis and anticancer activity of some novel thiazoles carrying 1,3,4-thiadiazole moiety

**DOI:** 10.1186/s13065-017-0255-7

**Published:** 2017-03-21

**Authors:** Sobhi M. Gomha, Nabila A. Kheder, Mohamad R. Abdelaziz, Yahia N. Mabkhot, Ahmad M. Alhajoj

**Affiliations:** 1 Department of Chemistry, Faculty of Science, Cairo University, Giza, 12613 Egypt; 2 Department of Pharmaceutical Chemistry, Faculty of Pharmacy, King Khalid University, Abha, 61441 Saudi Arabia; 3 Department of Pharmaceutical Chemistry, Faculty of Pharmacy, MIU University, Cairo, Egypt; 4 Department of Chemistry, College of Science, King Saud University, 2455, Riyadh, 11451 Saudi Arabia; 5 Department of Pharmacology, Faculty of Pharmacy, King Khalid University, Abha, 61441 Saudi Arabia

**Keywords:** Thiazoles, 1,3,4-Thiadiazoles, Hydrazonoyl chlorides, Anticancer activity, Structure activity relationship

## Abstract

**Background:**

Thiazoles and 1,3,4-thiadiazoles have been reported to possess various pharmacological activities.

**Results:**

A novel series of thiazoles carrying 1,3,4-thiadiazole core were designed and prepared via the reaction of the 2-(4-methyl-2-phenylthiazole-5-carbonyl)-*N*-phenylhydrazinecarbo-thioamide with the appropriate hydrazonoyl chlorides. The structures of the newly synthesized compounds were confirmed based on elemental and spectral analysis as well as their alternative syntheses. The cytotoxic potency of the newly synthesized thiadiazoles was evaluated by their growth inhibitory potency in liver HepG2 cancer cell line. Also, the structure activity relationship was studied.

**Conclusions:**

All the newly synthesized compounds were evaluated for their anticancer activity against liver carcinoma cell line (HepG2) using MTT assay. The results revealed that the compounds **12d**, **12c**, **6g**, **18b**, **6c**, and **6f** (IC50 = 0.82, 0.91, 1.06, 1.25, 1.29 and 1.88 µM, respectively) had good antitumor activity against liver carcinoma cell line (HepG2) when compared with the standard drug Doxorubicin (IC_50_ = 0.72 µM).

**Graphical abstract:**
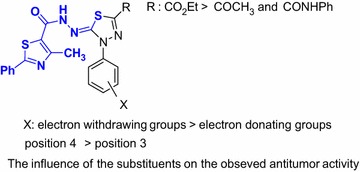
A facile synthesis and anticancer activity of some novel thiazoles carrying 1,3,4-thiadiazole moiety.

**Electronic supplementary material:**

The online version of this article (doi:10.1186/s13065-017-0255-7) contains supplementary material, which is available to authorized users.

## Background

Cancer is the most common life-threatening disease representing a major health problem for many decades. The clinical application of chemotherapy still considered as a major compartment in treating cancer, however, it is often limited by the severity of the side effects and the development of tumor cell resistance to these cytotoxic agents. Clinical administration of high doses of anticancer drugs to overcome resistance leads to severe toxicities [1]. Therefore, the development of novel effective anticancer drugs and strategies is eagerly being pursued.

Also, it was reported that liver cancer is one from the top ten human cancers worldwide and among the top five of cancers in terms of mortality [2, 3]. A literature survey revealed that thiazole derivatives had many biological activities as antihypertension [4], antifungal [5], antimicrobial [6, 7], anti-inflammatory [8], antioxidant [9], antitubercular [10], and anticancer [11–14]. Moreover, 1,3,4-thiadiazole derivatives had many biological activities such as antibacterial, antifungal, antituberculosis, anti-hepatitis B viral, antileishmanial, anti-inflammatory, analgesic, CNS depressant, antioxidant, antidiabetic, molluscicidal, antihypertensive, diuretic, analgesic, antimicrobial, antitubercular, anticonvulsant and anticancer [15–24]. These important biological activities encouraged several researchers to find out different methods for synthesis of new thiadiazoles using different synthons, such as thiosemicarbazides, thiocarbazides, dithiocarbazates, thioacylhydrazines, acyl hydrazines, and bithioureas [25]. As a part of our research projects to synthesize new bioactive compounds [26–34], we intended in this research to synthesize a new series of thiazoles carrying 1,3,4-thiadiazole moiety in order to study their anticancer activity against liver carcinoma cell line (HepG2).

## Results and discussion

### Chemistry

**Scheme 1:**
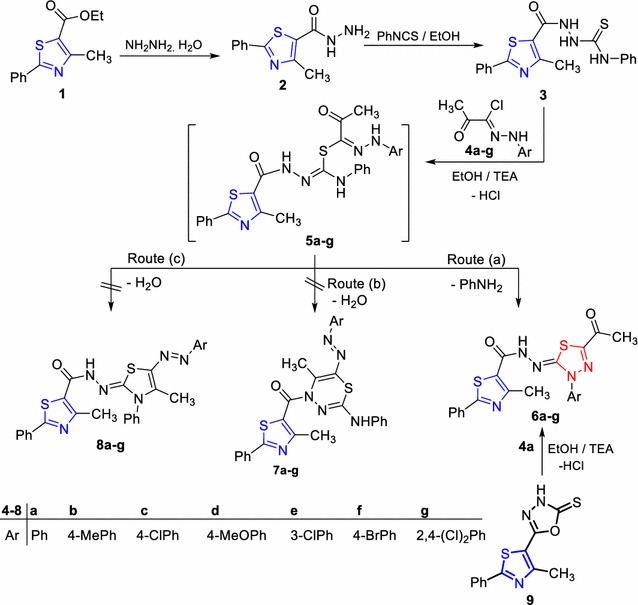
Synthesis of thiadiazoles **6a**–**g**

2-(4-Methyl-2-phenylthiazole-5-carbonyl)-*N*-phenylhydrazinecarbothioamide (**3**) [35] was prepared via reaction of 4-methyl-2-phenylthiazole-5-carbohydrazide (**2**) with phenyl isothiocyanate in ethanol (EtOH) as depicted in Scheme 1.

The presence of the thioamide hydrazine moiety as a side chain in compound **3** prompted us to utilize it for constructing 1,3,4-thiadiazole ring through its reaction with many hydrazonoyl chlorides. Thus, treatment of compound **3** with the appropriate hydrazonoyl chlorides **4a**–**g** [36] led to the formation of the respective 1,3,4-thiadiazoles **6a**–**g**, rather than thiadiazines **7a**–**g** or 1,3-thiazoles **8a**–**g** (Scheme 1). The elemental analysis together with the spectral data are consistent with the proposed structure **6**. The IR spectra of products **6** showed in each case the presence of two absorption bands around 1700, 1650 cm^−1^ for the two carbonyl groups, in addition to another band near v 3350 cm^−1^ for the NH function. The ^1^HNMR spectra of **6** showed in each case the presence of broad singlet signals assigned for the NH proton near δ 11.19 ppm, in addition to the expected signals for the two CH_3_, and the aryl protons. Also, the mass spectrum of each of products **6** revealed the presence of a molecular ion peak (see materials and methods). A suggested mechanism for the synthesis of 1,3,4-thiadiazole derivatives **6** is outlined in Scheme 1.

To explain the synthesis of 1,3,4-thiadiazole **6a**–**g**, we assumed that the reaction started with S-alkylation to afford the non-isolable intermediate **5** followed by intramolecular cyclization and elimination of aniline molecule to give the respective thiadiazole derivatives **6a**–**g** (Scheme 1). The structure of **6** was proved chemically via an alternative method (Scheme 1). Thus, the reaction of 5-(4-methyl-2-phenylthiazol-5-yl)-1,3,4-oxadiazole-2(3*H*)-thione (**9**) [37] with **4a** in ethanol in the presence of triethylamine under reflux led to the formation of a product which is identical in all respects (mp, mixed mp, and IR) with compound **6a**.

**Scheme 2:**
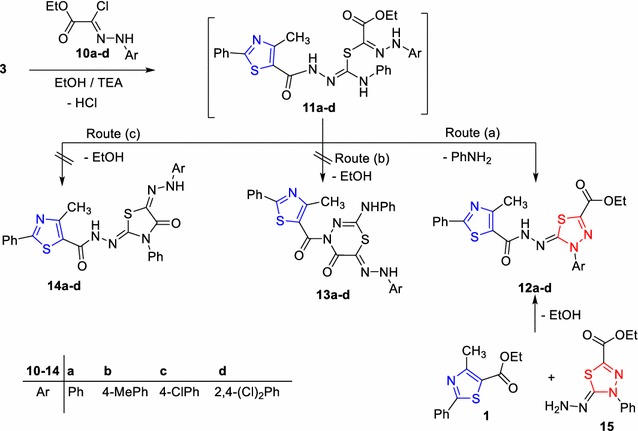
Synthesis of thiadiazole derivatives **12a**–**d**

Next, to test of the biological activities of a vast array of these compounds, we reacted compound **3** with the appropriate hydrazonoyl chlorides **10a**–**d** [38], under the same experimental conditions, which gave the corresponding 1,3,4-thiadiazole derivatives **12a**–**d** (Scheme 2). The IR and ^1^H-NMR spectra of **12a** taken as an example of the prepared series revealed the presence of the ester group and the disappearance of the hydrazone-NH function. Also, the mass spectrum of the reaction products **12a**–**d** showed, in each case, a peak corresponding to their molecular ions. The structure assigned for product **12** was further evidenced via an alternative method. Thus, the reaction of ethyl 4-methyl-2-phenyl thiazole-5-carboxylate (**1**) with 1,3,4-thiadiazole **15** [37] in ethanol under reflux, gave a product which was typical in all respects (mp, mixed mp, and IR) with that obtained from the reaction of **3** with **10a** (Scheme 2). To account for the formation of the product **12**, it was suggested that the reaction of compound **3** with hydrazonoyl chloride **10** initially gave the intermediate **11**, which underwent nucleophilic addition, followed by cyclization via losing of aniline molecule (route a) to give the final product **12**. The other routes (b) and (c) outlined in Scheme 2 were excluded since they led to the formation of products **13** and **14**, which were completely different in all respects (IR, ^1^H-NMR, mass spectra) from products **12**.

**Scheme 3:**
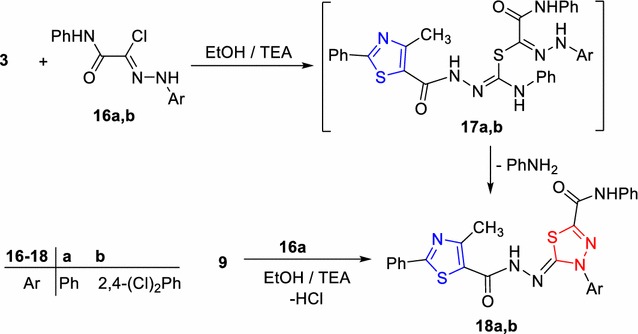
Synthesis of thiadiazole derivatives **18a**, **b**

Moreover, the reaction of compound **3** with hydrazonoyl halide of type **16** was studied. Thus refluxing compound **3** with the hydrazonoyl chloride **16a** or **16b** [38] under the same experimental conditions, afforded the corresponding 1,3,4-thiadiazole derivatives **18a**, **b** (Scheme 3). The ^1^H-NMR spectrum of compound **18a** revealed two D_2_O-exchangeable signals at δ 10.18 and 11.72 corresponding to two NH protons, in addition to an aromatic multiplet in the region 7.02–7.78 ppm. Also, its mass spectrum revealed a molecular ion peak at m/z = 512. In addition, compound **18a** was proved chemically via an alternative method from the reaction of compound **9** with **16a** which gave a product identical in all respects (mp, mixed mp, and IR) with compound **18a**.

### Cytotoxic activity

**Fig. 1:**
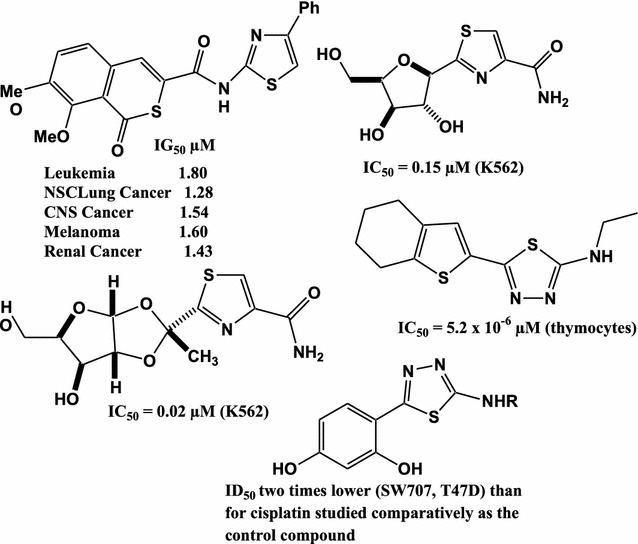
Antitumor activity of thiazoles and 1,3,4-thiadiazoles

The Literature survey showed that many 1,3-thiazole and 1,3,4-thiadiazole derivatives have antitumor activity with excellent IG_50_ and IC_50_ as depicted in Fig. 1 [38–44].

In view of these facts, the antitumor activity of the synthesized compounds was determined against liver carcinoma cell line HepG2. Doxorubicin was used as a reference standard. Data generated were used to plot a dose–response curve of which the concentration (μM) of test compounds required to kill 50% of the cell population (IC_50_) was determined. Cytotoxic activity was expressed as the mean IC_50_ of three independent experiments. The results depicted in Table 1.

**Table 1:** Cytotoxic activity of the tested compounds against HepG2

Sample number	R	X	IC_50_ (μM)
Doxorubicin	–	–	0.72 ± 0.13
**6a**	Ac	H	9.89 ± 0.19
**6b**	Ac	4-Me	39.06 ± 0.24
**6c**	Ac	4-Cl	1.29 ± 0.27
**6d**	Ac	4-OMe	64.35 ± 0.14
**6e**	Ac	3-Cl	4.03 ± 0.19
**6f**	Ac	4-Br	1.88 ± 0.08
**6g**	Ac	2,4-Cl_2_	1.06 ± 0.12
**12a**	CO_2_Et	H	4.70 ± 0.16
**12b**	CO_2_Et	4-Me	32.4 ± 0.19
**12c**	CO_2_Et	4-Cl	0.91 ± 0.20
**12d**	CO_2_Et	2,4-Cl_2_	0.82 ± 0.13
**18a**	CONHPh	H	6.79 ± 0.11
**18b**	CONHPh	2,4-Cl_2_	1.25 ± 0.18

**Fig. 2:**
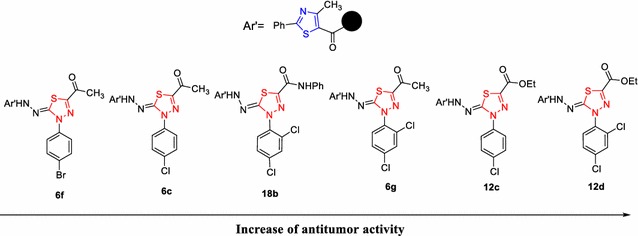
The influence of the substituents on the antitumor activity

The results of Table 1 revealed that most of the tested compounds showed a great variable activity compared to reference drug. The order of their antitumor activity and the influence of the substituents were shown in Fig. 2.

From the results of Table 1 and Fig. 2, we can suggest the following points.

- The ester group (CO_2_Et) at position 2 of the thiadiazole ring is necessary to have higher antitumor activity than the acetyl and the *N*-phenyl carboxamide (CONHPh) groups.
- The presence of chlorine group (electron-withdrawing group) at the position 2, 4 or 4 in the aryl moiety of the thiadiazole ring increased the cytotoxic activity.
- Chlorine at positions 2, 4 or 4 in the aryl moiety had high cytotoxic activity than halogen at position 3.
- The compounds containing chlorine had high cytotoxic activity than the compounds containing bromine.
- The presence of electron-donating groups such as methyl or methoxy at the position 4 in the aryl moiety as in the compounds **12b**, **6b** and **6d** decreased the cytotoxic activity.

## Experimental

### Chemistry

#### General

Melting points were measured on an Electrothermal IA 9000 series digital melting point apparatus (Bibby Sci. Lim. Stone, Staffordshire, UK). IR spectra were measured on PyeUnicam SP 3300 and Shimadzu FTIR 8101 PC infrared spectrophotometers (Shimadzu, Tokyo, Japan) in potassium bromide discs. NMR spectra were measured on a Varian Mercury VX-300 NMR spectrometer (Varian, Inc., Karlsruhe, Germany) operating at 300 MHz (^1^H-NMR) and run in deuterated dimethylsulfoxide (DMSO-*d*_*6*_). Chemical shifts were related to that of the solvent. Mass spectra were recorded on a Shimadzu GCMS-QP1000 EX mass spectrometer (Tokyo, Japan) at 70 eV. Elemental analyses were measured by using a German made Elementar vario LIII CHNS analyzer. Antitumor activity of the products was measured at the Regional Center for Mycology and Biotechnology at Al-Azhar University, Cairo, Egypt. 2-(4-Methyl-2-phenylthiazole-5-carbonyl)-*N*-phenylhydrazinecarbo-thioamide (**3**) [37], 5-(4-methyl-2-phenylthiazol-5-yl)-1,3,4-oxadiazole-2(3*H*)-thione (**9**) [37], hydrazonoyl halides **4a**–**g**, **10a**–**d** and **16a**, **b** [38], and ethyl 5-hydrazono-4-phenyl-4,5-dihydro-1,3,4-thiadiazole-2-carboxylate (**15**) [37] were prepared as reported in the respective literature.

#### Synthetic procedures

##### Synthesis of 1,3,4-thiadiazole derivatives (**6a**–**g**, **12a**–**d** and **18a**, **b**)

*General procedure* A mixture of compound **3** (0.368 g, 1 mmol) and the appropriate hydrazonoyl chlorides **4a**–**g** or **10a**–**d** or **16a**, **b** (1 mmol) in ethanol (20 mL), triethylamine (0.1 g, 1 mmol) was added. The mixture was refluxed for 4–6 h. The formed solid product was filtered, washed with methanol, dried and recrystallized from the proper solvents to afford products **6a**–**g**, **10a**–**d** and **18a**, **b**, respectively. The physical constants and spectral data of the obtained products are listed below:

###### *N′*-(5-Acetyl-3-phenyl-1,3,4-thiadiazol-2(3*H*)-ylidene)-4-methyl-2-phenyl thiazole-5-carbohydrazide (**6a**)

Yellow solid (73%); m.p. 163–165 °C (EtOH); IR (KBr) *v* 3317 (NH), 3038, 2951 (CH), 1701, 1647 (2C=O), 1593 (C=N) cm^−1^; ^1^H-NMR (DMSO-*d*_6_) *δ* 2.44 (s, 3H, CH_3_CO), 2.74 (s, 3H, CH_3_–thiazole), 6.92–8.00 (m, 10H, ArH), 11.19 (s, br, 1H, D_2_O-exchangeable NH); ^13^C-NMR (DMSO-*d*_6_): *δ* 16.9, 24.9 (CH_3_), 114.8, 117.1, 120.9, 121.9, 123.4, 126.2, 128.9, 129.2, 129.4, 130.9, 138.3, 141.7, 159.4 (Ar–C and C=N), 167.9, 194.0 (C=O); MS *m/z* (%) 435 (M^+^, 10), 381 (13), 274 (56), 118 (31), 92 (100), 65 (38). Anal. Calcd. for C_21_H_17_N_5_O_2_S_2_ (435.52): C, 57.91; H, 3.93; N, 16.08. Found C, 57.86; H, 3.84; N, 16.00%.

###### *N′*-(5-Acetyl-3-(*p*-tolyl)-1,3,4-thiadiazol-2(3*H*)-ylidene)-4-methyl-2-phenyl thiazole-5-carbohydrazide (**6b**)

Yellow solid (75%); m.p. 149–151 °C (EtOH); IR (KBr) *v* 3334 (NH), 3019, 2920 (CH), 1699, 1648 (2C=O), 1597 (C=N) cm^−1^; ^1^H-NMR (DMSO-*d*_6_) *δ* 2.31 (s, 3H, CH_3_-Ar), 2.44 (s, 3H, CH_3_CO), 2.73 (s, 3H, CH_3_–thiazole), 6.98–7.89 (m, 9H, ArH), 11.18 (s, br, 1H, D_2_O-exchangeable NH); ^13^C-NMR (DMSO-*d*_6_): *δ* 16.0, 17.7, 19.4 (CH_3_), 116.0, 118.0, 120.8, 125.1, 126.7, 127.3, 128.1, 129.8, 131.9, 132.7, 138.2, 152.6, 159.4 (Ar–C and C=N), 166.5, 194.7 (C=O); MS *m/z* (%) 449 (M^+^, 45), 372 (54), 200 (27), 104 (36), 80 (100), 64 (35). Anal. Calcd. for C_22_H_19_N_5_O_2_S_2_ (449.55): C, 58.78; H, 4.26; N, 15.58. Found C, 58.65; H, 4.17; N, 15.46%.

###### *N′*-(5-Acetyl-3-(4-chlorophenyl)-1,3,4-thiadiazol-2(3*H*)-ylidene)-4-methyl-2-phenyl-thiazole-5-carbohydrazide (**6c**)

Brown solid (75%); m.p. 171–173 °C (EtOH); IR (KBr) *v* 3325 (NH), 3013, 2926 (CH), 1698, 1655 (2C=O), 1594 (C=N) cm^−1^; ^1^H-NMR (DMSO-*d*_6_) *δ* 2.45 (s, 3H, CH_3_CO), 2.76 (s, 3H, CH_3_–thiazole), 6.93–7.96 (m, 9H, ArH), 11.25 (s, br, 1H, D_2_O-exchangeable NH); ^13^C-NMR (DMSO-*d*_6_): *δ* 16.8, 24.9 (CH_3_), 115.1, 119.4, 120.2, 122.9, 123.8, 127.3, 128.3, 128.7, 129.0, 133.5, 138.3, 140.2, 157.9 (Ar–C and C=N), 167.8, 194.3 (C=O); MS *m/z* (%) 471 (M^+^+2, 14), 469 (M^+^, 45), 396 (57), 200 (17), 80 (100), 64 (89). Anal. Calcd. for C_21_H_16_ClN_5_O_2_S_2_ (469.97): C, 53.67; H, 3.43; N, 14.90. Found C, 53.52; H, 3.37; N, 14.82%.

###### *N′*-(5-Acetyl-3-(4-methoxyphenyl)-1,3,4-thiadiazol-2(3*H*)-ylidene)-4-methyl-2-phenyl-thiazole-5-carbohydrazide (**6d**)

Brown solid (68%); m.p. 143–145 °C (EtOH); IR (KBr) *v* 3328 (NH), 3031, 2923 (CH), 1697, 1653 (2C=O), 1596 (C=N) cm^−1^; ^1^H-NMR (DMSO-*d*_6_) *δ* 2.45 (s, 3H, CH_3_CO), 2.75 (s, 3H, CH_3_–thiazole), 3.76 (s, 3H, OCH_3_), 6.99–7.99 (m, 9H, ArH), 11.29 (s, br, 1H, D_2_O-exchangeable NH); ^13^C-NMR (DMSO-*d*_6_): *δ* 16.5, 17.9, 54.2 (CH_3_), 116.2, 117.9, 120.7, 124.8, 126.3, 127.0, 127.7, 129.3, 131.9, 132.4, 137.6, 150.2, 159.0 (Ar–C and C=N), 166.2, 194.6 (C=O); MS *m/z* (%) 465 (M^+^, 39), 334 (87), 200 (63), 122 (80), 77 (100), 64 (45). Anal. Calcd. for C_22_H_19_N_5_O_3_S_2_ (465.55): C, 56.76; H, 4.11; N, 15.04. Found C, 56.63; H, 4.04; N, 14.95%.

###### *N′*-(5-Acetyl-3-(3-chlorophenyl)-1,3,4-thiadiazol-2(3*H*)-ylidene)-4-methyl-2-phenylthiazole-5-carbohydrazide (**6e**)

Yellow solid (70%); m.p. 166–168 °C (EtOH); IR (KBr) *v* 3431(NH), 3025, 2932 (CH), 1698, 1659 (2C=O), 1593 (C=N) cm^−1^; ^1^H-NMR (DMSO-*d*_6_) *δ* 2.44 (s, 3H, CH_3_CO), 2.66 (s, 3H, CH_3_–thiazole), 6.98–7.90 (m, 9H, ArH), 11.23 (s, br, 1H, D_2_O-exchangeable NH); MS *m/z* (%) 471 (M^+^+2, 10), 469 (M^+^, 34), 334 (46), 200 (28), 132 (48), 80 (100), 64 (68). Anal. Calcd. for C_21_H_16_ClN_5_O_2_S_2_ (469.97): C, 53.67; H, 3.43; N, 14.90. Found C, 53.60; H, 3.36; N, 14.79%.

###### *N′*-(5-Acetyl-3-(4-bromophenyl)-1,3,4-thiadiazol-2(3*H*)-ylidene)-4-methyl-2-phenyl thiazole-5-carbohydrazide (**6f**)

Brown solid (73%); m.p. 160–162 °C (EtOH); IR (KBr) *v* 3429 (NH), 3012, 2924 (CH), 1696, 1654 (2C=O), 1594 (C=N) cm^−1^; ^1^H-NMR (DMSO-*d*_6_) *δ* 2.44 (s, 3H, CH_3_CO), 2.65 (s, 3H, CH_3_–thiazole), 6.95–7.94 (m, 9H, ArH), 11.25 (s, br, 1H, D_2_O-exchangeable NH); ^13^C-NMR (DMSO-*d*_6_): *δ* 16.9, 24.8 (CH_3_), 114.8, 120.3, 122.0, 122.6, 123.8, 127.2, 127.9, 128.3, 130.2, 132.5, 136.9, 140.0, 157.5 (Ar–C and C=N), 167.6, 194.1 (C=O); MS *m/z* (%) 516 (51), 514 (M^+^, 53), 325 (76), 172 (44), 91 (80), 80 (100), 64 (47). Anal. Calcd. for C_21_H_16_BrN_5_O_2_S_2_ (514.42): C, 49.03; H, 3.14; N, 13.61. Found C, 48.93; H, 3.12; N, 13.53%.

###### *N′*-(5-Acetyl-3-(2,4-dichlorophenyl)-1,3,4-thiadiazol-2(3*H*)-ylidene)-4-methyl-2-phenyl thiazole-5-carbohydrazide (**6g**)

Brown solid (77%); m.p. 181–183 °C (EtOH/dioxane); IR (KBr) *v* 3318 (NH), 3088, 2926 (CH), 1699, 1671 (2C=O), 1597 (C=N) cm^−1^; ^1^H-NMR (DMSO-*d*_6_) *δ* 2.47 (s, 3H, CH_3_CO), 2.67 (s, 3H, CH_3_–thiazole), 6.97–8.07 (m, 8H, ArH), 11.19 (s, br, 1H, D_2_O-exchangeable NH); MS *m/z* (%) 504 (M^+^, 14), 407 (33), 161 (14), 80 (99), 64 (100). Anal. Calcd. for C_21_H_15_Cl_2_N_5_O_2_S_2_ (504.41): C, 50.00; H, 3.00; N, 13.88. Found C, 49.88; H, 2.92; N, 13.75%.

###### Ethyl 5-(2-(4-methyl-2-phenylthiazole-5-carbonyl)hydrazono)-4-phenyl-4,5-dihydro-1,3,4-thiadiazole-2-carboxylate (**12a**)

Yellow solid (71%); m.p. 137–139 °C (EtOH); IR (KBr) *v* 3432 (NH), 3035, 2923 (CH), 1749, 1659 (2C=O), 1597 (C = N) cm^−1^; ^1^H-NMR (DMSO-*d*_6_) *δ* 1.20 (t, 3H, *J* = 7.1 Hz, CH_2_CH_3_), 2.74 (s, 3H, CH_3_–thiazole), 4.21 (q, 2H, *J* = 7.1 Hz, CH_2_CH_3_),7.00–8.01 (m, 10H, ArH), 10.72 (s, br, 1H, D_2_O-exchangeable NH); ^13^C-NMR (DMSO-*d*_6_): *δ* 13.7, 16.8 (CH_3_), 61.2 (CH_2_), 115.8, 117.3, 118.4, 120.9, 122.4, 126.0, 128.5, 128.9, 130.0, 132.6, 135.6, 139.6, 159.1 (Ar–C and C=N), 163.4, 166.8 (C=O); MS *m/z* (%): 465 (M^+^, 27), 334 (50), 200 (34), 104 (40), 80 (100), 64 (37). Anal. Calcd. for C_22_H_19_N_5_O_3_S_2_ (465.55): C, 56.76; H, 4.11; N, 15.04. Found C, 56.69; H, 4.03; N, 15.01%.

###### Ethyl 5-(2-(4-methyl-2-phenylthiazole-5-carbonyl)hydrazono)-4-(*p*-tolyl)-4,5-dihydro-1,3,4-thiadiazole-2-carboxylate (**12b**)

Yellow solid (70%); m.p. 147–149 °C (EtOH); IR (KBr) *v* 3424 (NH), 3058, 2925 (CH), 1749, 1674 (2C=O), 1595 (C=N) cm^−1^; ^1^H-NMR (DMSO-*d*_6_) *δ* 1.20 (t, 3H, *J* = 7.1 Hz, CH_2_CH_3_), 2.26 (s, 3H, CH_3_–Ar), 2.76 (s, 3H, CH_3_–thiazole), 4.19 (q, 2H, *J* = 7.1 Hz, CH_2_CH_3_), 7.00–8.02 (m, 9H, ArH), 10.73 (s, br, 1H, D_2_O-exchangeable NH); ^13^C-NMR (DMSO-*d*_6_): *δ* 13.9, 16.8, 20.1 (CH_3_), 61.5 (CH_2_), 114.5, 115.8, 117.1, 120.9, 121.9, 126.2, 128.1, 129.6, 130.8, 131.8, 138.3, 140.0, 159.4 (Ar–C and C=N), 163.0, 166.5 (C=O); MS *m/z* (%) 479 (M^+^, 20), 367 (25), 251 (18), 80 (85), 64 (100). Anal. Calcd. for C_23_H_21_N_5_O_3_S_2_ (479.57): C, 57.60; H, 4.41; N, 14.60. Found C, 57.49; H, 4.33; N, 14.51%.

###### Ethyl 4-(4-chlorophenyl)-5-(2-(4-methyl-2-phenylthiazole-5-carbonyl) hydrazono)-4,5-dihydro-1,3,4-thiadiazole-2-carboxylate (**12c**)

Yellow solid (73%); m.p. 167–169 °C (EtOH/dioxane); IR (KBr) *v* 3340 (NH), 3050, 2927 (CH), 1748, 1670 (2C=O), 1599 (C=N) cm^−1^; ^1^H-NMR (DMSO-*d*_6_) *δ* 1.23 (t, 3H, *J* = 7.1 Hz, CH_2_CH_3_), 2.75 (s, 3H, CH_3_–thiazole), 4.22 (q, 2H, *J* = 7.1 Hz, CH_2_CH_3_),7.02–7.96 (m, 9H, ArH), 10.77 (s, br, 1H, D_2_O-exchangeable NH); ^13^C-NMR (DMSO-*d*_6_): *δ* 13.4, 16.9 (CH_3_), 61.4 (CH_2_), 116.2, 117.0, 119.5, 120.9, 122.3, 127.2, 128.2, 129.4, 131.4, 132.2, 137.0, 139.4, 158.6 (Ar–C and C=N), 163.8, 167.2 (C=O); MS *m/z* (%) 501 (M^+^+2, 13), 499 (M^+^, 45), 363 (39), 334 (100), 200 (35), 104 (30), 77 (50). Anal. Calcd. for C_22_H_18_ClN_5_O_3_S_2_ (499.99): C, 52.85; H, 3.63; N, 14.01. Found C, 52.79; H, 3.60; N, 13.87%.

###### Ethyl 4-(2,4-dichlorophenyl)-5-(2-(4-methyl-2-phenylthiazole-5-carbonyl) hydrazono)-4,5-dihydro-1,3,4-thiadiazole-2-carboxylate (**12d**)

Brown solid (75%); m.p. 173–175 °C (EtOH/dioxane); IR (KBr) *v* 3221 (NH), 3079, 2926 (CH), 1749, 1671 (2C=O), 1599 (C=N) cm^−1^; ^1^H-NMR (DMSO-*d*_6_) *δ* 1.24 (t, 3H, *J* = 7.1 Hz, CH_2_CH_3_), 2.77 (s, 3H, CH_3_-thiazole), 4.23 (q, 2H, *J* = 7.1 Hz, CH_2_CH_3_),7.08–8.13 (m, 8H, ArH), 10.77 (s, br, 1H, D_2_O-exchangeable NH); MS *m/z* (%) 534 (M^+^, 19), 449 (78), 223 (100), 200 (54), 104 (58), 80 (85). Anal. Calcd. for C_22_H_17_Cl_2_N_5_O_3_S_2_ (534.44): C, 49.44; H, 3.21; N, 13.10. Found C, 49.29; H, 3.16; N, 13.02%.

###### 5-(2-(4-Methyl-2-phenylthiazole-5-carbonyl)hydrazono)-*N*,4-diphenyl-4,5-dihydro-1,3,4-thiadiazole-2-carboxamide (**18a**)

Brown solid (76%); m.p. 176–178 °C (EtOH/dioxane); IR (KBr) *v* 3427, 3343 (2NH), 1672, 1653 (2C=O), 1597 (C=N) cm^−1^; ^1^H-NMR (DMSO-*d*_6_) *δ* 2.75 (s, 3H, CH_3_–thiazole), 7.02–7.78 (m, 15H, ArH), 10.18 (s, br, 1H, D_2_O-exchangeable NH), 11.72 (s, br, 1H, D_2_O-exchangeable NH); ^13^C-NMR (DMSO-*d*_6_): *δ* 17.2 (CH_3_), 114.6, 117.3, 118.4, 120.9, 122.6, 122.8, 124.0, 126.5, 128.5, 129.1, 130.0, 130.6, 131.9, 132.6, 138.2, 142.1, 159.2 (Ar–C and C=N), 162.6, 166.0 (C=O); MS *m/z* (%) 512 (M^+^, 8), 401 (00), 282 (10), 150 (22), 92 (26), 65 (29). Anal. Calcd. For C_26_H_20_N_6_O_2_S_2_ (512.61): C, 60.92; H, 3.93; N, 16.39. Found C, 60.78; H, 3.85; N, 16.32%.

###### 4-(2,4-Dichlorophenyl)-5-(2-(4-methyl-2-phenylthiazole-5-carbonyl) hydrazono)-*N*-phenyl-4,5-dihydro-1,3,4-thiadiazole-2-carboxamide (**18b**)

Brown solid (77%); m.p. 186–188 °C (Dioxane); IR (KBr) *v* 3429, 3337 (2NH), 1692, 1656 (2C=O), 1591 (C=N) cm^−1^; ^1^H-NMR (DMSO-*d*_6_) *δ* 2.76 (s, 3H, CH_3_–thiazole), 7.13–7.83 (m, 13H, ArH), 10.19 (s, br, 1H, D_2_O-exchangeable NH), 11.77 (s, br, 1H, D_2_O-exchangeable NH); MS *m/z* (%) 581 (M^+^, 38), 473 (64), 334 (72), 200 (35), 119 (65), 64 (100). Anal. Calcd. for C_26_H_18_Cl_2_N_6_O_2_S_2_ (581.50): C, 53.70; H, 3.12; N, 14.45. Found C, 53.62; H, 3.03; N, 14.32%.

##### Alternate synthesis of thiadiazole derivatives **6a** and **18a**

To a mixture of 5-(4-methyl-2-phenylthiazol-5-yl)-1,3,4-oxadiazole-2(3*H*)-thione (**9**) (0.275 g, 1 mmol) and hydrazonoyl chloride **4a** or **16a** (1 mmol) in absolute EtOH (25 mL), was added triethylamine (0.1 g, 0.14 mL, 1 mmol). The reaction mixture was stirred at room temperature till methyl mercaptan ceased to evolve (3 h). The solvent was evaporated and the residue was treated with ice/HCl mixture. The solid product was collected by filtration, washed with EtOH, dried, and recrystallized to give the respective compounds **6a** and **18a**, that was identical in all respects (m.p., mixed m.p. and IR spectra) with that obtained from the reaction of **4a** or **16a** with **3**.

##### Alternate synthesis of 12a

A mixture of ethyl 4-methyl-2-phenylthiazole-5-carboxylate (**1**) (0.247 g, 1 mmol) and ethyl 5-hydrazono-4-phenyl-4,5-dihydro-1,3,4-thiadiazole-2-carboxylate (**15**) (0.264 g, 1 mmol) was refluxed in ethanol for 4 h. The solid product that separated was filtered off, washed with water and finally recrystallized to give the corresponding product, **12a** which was identical in all aspects (m.p., mixed m.p. and IR spectra) with those obtained from the reaction of **3** with **10a**.

### Evaluation of the antitumor activity using Viability assay

Human hepatocellular carcinoma (HepG2) cell line was obtained from the American Type Culture Collection (ATCC, Rockville, MD). The detailed procedure for the in vitro antitumor assay is presented in Additional file 1.

## Conclusions

A series of novel thiazoles carrying 1,3,4-thiadiazole ring were synthesized. The structure of the newly prepared compounds was established based on both elemental analysis and spectroscopic data and by an alternative method wherever possible. All the synthesized compounds were evaluated for their anti-cancer activity against the human hepatocellular carcinoma (HepG2) cell line. The results showed that the thiazole derivatives **12d**, **12c**, **6g**,**18b**, **6c** and **6f** having IC_50_ values 0.82, 0.91, 1.06, 1.25, 1.29 and 1.88 µM, respectively, were found to be the highly active compounds of the prepared series. Based on the experimental results of the antitumor activity, the structure–activity relationships were discussed.

## Additional file

**Additional file 1.** Supporting informations.

## Abbreviations

HepG2: human hepatocellular carcinoma
SAR: structure activity relationship
EtOH: ethanol
m.p.: melting point
TEA: triethylamine
IR: infra-red
ATCC: American Type Culture Collection
TLC: thin layer chromatography
