# Supplementary material for: A facile synthesis and anticancer activity of some novel thiazoles carrying 1,3,4-thiadiazole moiety
Source: Chem Cent J. 2017 Mar 21;11:25. doi: 10.1186/s13065-017-0255-7 (PMC5360743; doi:10.1186/s13065-017-0255-7)
Supplement: Supplementary file 1 — Additional file 1. Supporting informations. [file 13065_2017_255_MOESM1_ESM.docx]

A Facile Synthesis and Anticancer Activity of Some Novel Thiazoles Carrying 1,3,4-Thiadiazole Moiety

Sobhi M. Gomha,^1,*^ Nabila A. Kheder,^1,2^ Mohamad R. Abdelaziz,^3^ Yahia N. Mabkhot,^4^ and Ahmad M. Alhajoj^5^

^1^Department of Chemistry, Faculty of Science, Cairo University, Giza 12613, Egypt

^2^Department of Pharmaceutical Chemistry, Faculty of Pharmacy, King Khalid University, Abha 61441, Saudi Arabia.

^3^Department of pharmaceutical Chemistry, Faculty of Pharmacy, MIU University, Egypt.

^4^Department of Chemistry, College of Science, King Saud University, P. O. Box 2455, Riyadh-11451, Saudi Arabia.

^5^Department of Pharmacology, Faculty of Pharmacy, King Khalid University, Abha 61441, Saudi Arabia.

**2.2. Cytotoxic activity**

Human hepatocellular carcinoma (HepG2) cell line was obtained from the American Type Culture Collection (ATCC, Rockville, MD). The cells were grown on RPMI-1640 medium supplemented with 10% inactivated fetal calf serum and 50 µg/mL gentamycin. The cells were maintained at 37 oC in a humidified atmosphere with 5% carbon dioxide and were subcultured 2 to 3 times a week. Potential cytotoxicity of the tested compounds was evaluated on tumor cells using the reported method of Gangadevi and Muthumary [1]. The cells were grown as monolayers in growth RPMI-1640. The monolayers of 104 cells adhered at the bottom of the wells in a 96-well microtiter plate incubated for 24 h at 37 oC in a humidified incubator with 5% carbon dioxide. The monolayers were then washed with sterile phosphate buffered saline (0.01 M pH 7.2) and simultaneously the cells were treated with 100 µL from different dilutions of tested sample in fresh maintenance medium and incubated at 37 oC. A control of untreated cells was made in the absence of tested sample. Positive controls containing doxroubcin drug was also tested as reference drug for comparison. Six wells were used for each concentration of the test sample. Every 24 h the observation under the inverted microscope was made.The number of the surviving cells was determined by staining the cells with crystal violet [2] followed by cell lysing using 33% glacial acetic acid and read the absorbance at 590 nm using microplate reader (SunRise, TECAN, Inc, USA) after well mixing. The absorbance values from untreated cells were considered as 100% proliferation. The number of viable cells was determined using microplate reader as previously mentioned before and the percentage of viability was calculated as [1-(ODt/ODc)] x 100% where ODt is the mean optical density of wells treated with the tested sample and ODc is the mean optical density of untreated cells. The relation between surviving cells and drug concentration is plotted to get the survival curve of each tumor cell line after treatment with the specified compound. The 50% inhibitory concentration (IC50), the concentration required to cause toxic effects in 50% of intact cells, was estimated from graphic plots.

1. Gangadevi, V.; Muthumary, Preliminary studies on cytotoxic effect of fungal taxol on cancer cell lines. J .African J. Biotech. 2007, 6, 1382-1686.
2. Mosmann, T. Rapid colorimetric assay for cellular growth and survival: Application to proliferation and cytotoxicity assays. J. Immunol. Methods 1983, 65, 55-63.
